# Improved Multi-Sensor Fusion Dynamic Odometry Based on Neural Networks

**DOI:** 10.3390/s24196193

**Published:** 2024-09-25

**Authors:** Lishu Luo, Fulun Peng, Longhui Dong

**Affiliations:** Xi’an Institute of Applied Optics, Xi’an 710065, China

**Keywords:** simultaneous localization and mapping (SLAM), multi-sensor fusion, dynamic elimination

## Abstract

High-precision simultaneous localization and mapping (SLAM) in dynamic real-world environments plays a crucial role in autonomous robot navigation, self-driving cars, and drone control. To address this dynamic localization issue, in this paper, a dynamic odometry method is proposed based on FAST-LIVO, a fast LiDAR (light detection and ranging)–inertial–visual odometry system, integrating neural networks with laser, camera, and inertial measurement unit modalities. The method first constructs visual–inertial and LiDAR–inertial odometry subsystems. Then, a lightweight neural network is used to remove dynamic elements from the visual part, and dynamic clustering is applied to the LiDAR part to eliminate dynamic environments, ensuring the reliability of the remaining environmental data. Validation of the datasets shows that the proposed multi-sensor fusion dynamic odometry can achieve high-precision pose estimation in complex dynamic environments with high continuity, reliability, and dynamic robustness.

## 1. Introduction

High-precision simultaneous localization and mapping (SLAM) plays a critical role in fields such as autonomous navigation for robots, self-driving cars, and drone control. Achieving high-precision SLAM requires accurate localization, meaning that the intelligent agent must know its exact position. Currently, precise localization methods widely rely on the Global Navigation Satellite System (GNSS). However, in complex urban environments with tall buildings and dense trees, the GNSS can encounter denied environments. Therefore, in GNSS-denied urban environments, SLAM requires high-precision localization methods that do not depend on the GNSS.

Odometry is a measurement method that uses sensor data to estimate pose changes over time. Traditional odometry relies on a single sensor, such as an inertial measurement unit (IMU), visual camera, or light detection and ranging (LiDAR). However, because of the sensors’ characteristics, single-sensor odometry is prone to significant cumulative localization errors [1]. As the number of sensors onboard intelligent agents increases and sensor technology advances, dual- and multi-sensor fusion odometry can combine the advantages of various sensors to achieve higher localization accuracy.

In two-sensor fusion odometry, the mainstream approaches are visual–inertial odometry (VIO) and LiDAR–inertial odometry (LIO), both utilizing the IMU’s advantages of fast data updates and high autonomy [2]. VIO is lightweight overall, primarily using feature matching to estimate the agent’s pose between frames. Researchers [3] have developed monocular and stereo visual–inertial SLAM systems using oriented FAST and rotated BRIEF (ORB) feature point extraction and matching techniques [4] to calculate camera pose changes between adjacent frames and improve pose estimation accuracy and robustness through loose coupling with IMU data. Authors in [5], building on the work of [3], performed a tight coupling of visual and IMU information using SuperPoint—a method based on convolutional neural networks—for feature point extraction and descriptor matching [6]. This approach enhances the accuracy and robustness of pose estimation. The authors of [7] employed sliding window optimization and factor graph optimization, demonstrating good performance in dynamic environments and high-speed motion.

Although VIO offers advantages such as lower device costs and higher autonomy and concealment, its performance can significantly degrade under limited lighting conditions because of its high sensitivity to environmental factors. However, LiDAR can better leverage its advantages to obtain more precise distance information in harsh environments [8], thus providing more accurate self-pose estimation. Research on LiDAR and its coupling with LIO is relatively thorough [9]. The studies [7,10] both used sliding window optimization methods to align each frame with a local map, ensuring that each frame established a clear constraint relationship with the first frame. The authors of [11], building on [10], constructed a local map by stitching together the latest keyframes and updated the local map based on pose information. Several studies [12,13,14] used iterative error Kalman filtering as a filtering framework, performing state updates through iterative solutions to achieve more precise pose estimation.

LiDAR can provide precise distance information with high resolution and reliability, but its performance deteriorates in dynamic environments and severely degrades in feature-poor environments, such as open plains or long, straight corridors. By combining visual cameras, LiDAR, and IMUs to form laser–visual–inertial odometry (LVIO), the advantages of each sensor can be leveraged to effectively mitigate the limitations of individual sensors.

Authors in [14] constructed a tightly coupled factor graph model that integrates measurements from visual cameras, LiDAR, and IMUs, treating primitives as landmarks and tracking them across multiple scans, thereby enhancing robustness in complex scenarios. Researchers in [15] built on the LIO system from [13] and the VIO system from [7], using a tight coupling method to combine both systems and incorporating high-frequency odometry filtering. This approach enabled the proposed algorithm to be used for dense 3D mapping over large indoor and outdoor areas. The work in [16,17], based on [15], continued using the LIO component, separating VIO into inter-frame tracking and frame-to-map tracking, and updating the iterative extended Kalman filtering process to achieve more accurate map construction.

However, in the complex urban environment, city roads are filled with numerous moving vehicles and pedestrians. Achieving high-precision SLAM in dynamic environments remains a significant challenge for current LVIO algorithms, as the movement of environmental objects can lead to the misidentification of surrounding obstacles and severely impact keyframe extraction and inter-frame tracking. To adapt to real-world dynamic environments, we consider the excellent performance of neural networks in dynamic object recognition [18,19]. Traditional neural network structures are complex, computationally demanding, and difficult to integrate with SLAM algorithms. Lightweight neural networks balance dynamic recognition performance with computational efficiency, and combining these networks with LVIO algorithms can achieve high-precision SLAM in dynamic and complex environments.

## 2. Multi-Sensor Fusion Dynamic Odometry Calculation Framework

**Figure 1:**
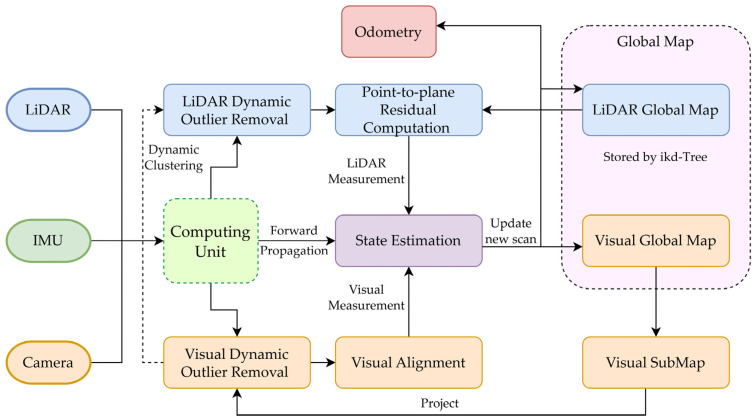
Multi-sensor fusion dynamic odometry calculation framework diagram.

The multi-sensor fusion dynamic odometry architecture presented in this paper references the FAST-LIVO algorithm [17]. FAST-LIVO is a fast multi-sensor fusion odometry system that primarily consists of LIO and VIO subsystems. In the preprocessing phase, sensor information within a certain time frame is packaged into computational units. This phase includes anomaly removal from visual information and dynamic object removal based on a lightweight neural network. During the state estimation phase, iterative Kalman filtering is applied through LiDAR point-to-plane residual calculation, IMU prior estimation, and camera sparse-direct visual alignment. A local map and an ikd-Tree [20] global map are built simultaneously. Additionally, the dynamic parts of the radar point cloud corresponding to the dynamic segments removed from visual information are also removed through dynamic clustering. The filtered global map information is then used in the LiDAR point-to-plane residual calculation. The specific system algorithm framework is shown in Figure 1.

## 3. System Description

### 3.1. State Transition Model

The state transition model, also known as the discrete model, describes the changes in the system state over discrete time intervals. In this system, we assume that the LiDAR, camera, and IMU have been pre-calibrated and synchronized, and that the time drift between the three sensors is known. The state transition model is primarily based on IMU data. By combining the measured angular velocity and acceleration and removing their respective biases and noise, we obtain the net values. We assume that the three sensors are rigidly connected. Equation (1) defines the state transition model at the moment i when the IMU measurement is taken:

(1) $$x_{i+1}=x_{i}⊞\left(\Delta tf\left(x_{i},u_{i},w_{i}\right)\right)$$

where **x***_i_* represents the system state vector at time step *i*, ⊞ represents generalized addition, Δt is the time step, **u***_i_* is the control input at time step *i*, **w***_i_* is the process noise at time step *i,* and $f\left(x,u,w\right)$ is the state transition function. Equation (2) provides detailed definitions for x, u, w, and $f\left(x,u,w\right)$:

(2) M≜SO(3)×ℝ15,dim(M)=18x≜RITGpITGvITGbgTbaTgTGT∈Mu≜ωmTamTT,w≜ngTnaTnbgTnbaTTfxi,ui,wi=ωmi−bgi−nωivIiG+12RIiGami−bai−nai+GgiΔtRIiGami−bai−nai+Gginbginbai03×1

where the manifold M is an 18-dimensional composite manifold composed of the 3-dimensional rotation group SO(3) and a 15-dimensional free Euclidean space R^15^, the body coordinate system *I* is the IMU’s coordinate system, and the global coordinate system G is established with reference to the first frame of the IMU coordinate system. RIG, pIG, and vIG represent the attitude, position, and velocity in the global coordinate system, respectively. $a_{m}$ and $\omega_{m}$ are the acceleration and angular velocity, respectively, measured by the IMU. $n_{a}$ and $n_{g}$ are the white noises of $a_{m}$ and $\omega_{m}$, respectively. $n_{ba}$ and $n_{bg}$ represent the Gaussian noises, while $b_{a}$ and $b_{g}$ are the random walk processes of $n_{ba}$ and $n_{bg}$, respectively.

### 3.2. Kinematic Model

The kinematic model, also known as the continuous model, describes the changes in the system state over continuous time. In Section 3.1, we assume that the three sensors are rigidly connected. Through joint calibration, the extrinsic parameter matrix TLI=RLI,pLI from the LiDAR to the IMU can be obtained, where *L* denotes the LiDAR coordinate system. Equation (3) provides a detailed definition of the kinematic model:

(3) p˙IG=vIG,v˙IG=RIGam−ba−na+gG,g˙G=0R˙IG=RIGωm−bω−nω∧,b˙g=nbg,b˙a=nba

where ${\left\lfloor \omega_{m}-b_{\omega }-n_{\omega }\right\rfloor }_{\wedge }$ represents the antisymmetric model of vector $\omega_{m}-b_{\omega }-n_{\omega }$, mapping the cross-product operation to the dot-product operation. The kinematic model combines the IMU’s acceleration and angular velocity measurements, accurately describing the changes in the IMU’s attitude, velocity, and position, thus providing the foundation for subsequent state estimation.

### 3.3. State Estimation Model

To estimate the state x in Equation (1), we use the iterative extended Kalman filter (IEKF), as detailed in Section 3.4. The IEKF iteratively estimates and updates the system state through nonlinear state transition and observation equations. We define the end time of the previous LiDAR or camera scan as ***t***_*k*−1_, at which the best state estimate of $x_{k-1}$ is ${\bar{x}}_{k-1}$, and the corresponding covariance matrix is ${\bar{P}}_{k-1}$. The state error vector is defined by Equation (4):

(4) x˜k−1≜xk−1⊟x¯k−1=δθTp˜ITGv˜ITGb˜gTb˜aTg˜TGT

where ${\tilde{x}}_{k-1}$ is the error vector of $x_{k-1}$, the prime symbol ⊟ represents generalized subtraction, and $\delta \theta =\mathrm{Log}{(}^{G}{\bar{R}}_{I}^{TG}R_{I})$ is the attitude error, describing the slight deviation between the true and estimated attitudes.

#### 3.3.1. Forward Propagation

The state is propagated through Equation (1). Forward propagation begins upon receiving the first frame of IMU data. By setting the process noise $w_{i}$ to 0, the forward propagation in Equation (5) can be obtained:

(5) $$\begin{array}{ll} {\tilde{x}}_{i+1} & =x_{i+1}⊞{\hat{x}}_{i+1}\\ & =(x_{i}⊞\Delta tf\left(x_{i},u_{i},w_{i}\right))⊞({\hat{x}}_{i}⊞\Delta tf\left({\hat{x}}_{i},u_{i},0\right))\\ & ≃F_{\tilde{x}}{\tilde{x}}_{i}+F_{w}w_{i} \end{array}$$

where $F_{\tilde{x}}$ and $F_{w}$ are the Jacobian matrices of the error state vector ${\tilde{x}}_{i}$ and the process noise $w_{i}$, respectively. These are obtained by taking the partial derivatives of the state transition function f(∙) with respect to ${\tilde{x}}_{i}$ and $w_{i}$, respectively. This allows the state estimation model to better handle nonlinear systems, describing how the state changes depend on the current state and system process noise. According to Equation (6), the covariance propagation formula and the propagation initialization conditions can be defined:

(6) $${\hat{P}}_{i+1}=F_{\tilde{x}}{\hat{P}}_{i}F_{\tilde{x}}^{T}+F_{w}QF_{w}^{T};{\hat{P}}_{0}={\bar{P}}_{k-1}$$

where Q is the covariance matrix of the process noise w. Time $t_{k}$ is noted as the time when the current LiDAR or camera scan is received. Equations (5) and (6) constitute the state update for the LiDAR or camera.

#### 3.3.2. LIO Data Alignment

**Figure 2:**
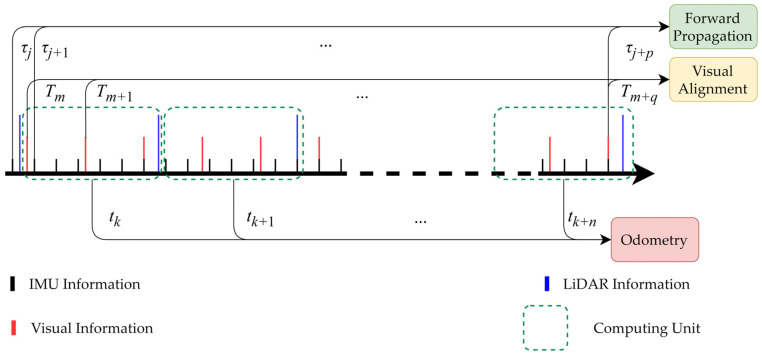
LIO data alignment diagram.

This study improves data alignment in the LIO section relative to [18], as shown in Figure 2. The LiDAR’s scanning frequency is lower than that of the visual camera and significantly lower than that of the IMU. All sensor data within $\left.\left(t_{k}\right.,t_{k+1}\right]$ are packaged into a computational unit. The data within the computational unit are processed in chronological order. When processing IMU data at time $\tau_{j}\in \left.\left(t_{k}\right.,t_{k+1}\right]$, forward propagation is performed using Kalman filtering. When processing image data at time $T_{m}\in \left.\left(t_{k}\right.,t_{k+1}\right]$, a direct visual residual updating system state is constructed. When processing LiDAR data at time $t_{k+1}$, the most recent image data are used to remove dynamic points. Then, a point cloud registration residual is established and used to update the system state.

#### 3.3.3. LiDAR Measurement Model

When registering these scan points ${\{}^{L}p_{j}\}$ to the map, assuming each point is on the adjacent planes in the map, with the normal vector and center point of the planes being $u_{j}$ and $q_{j}$, respectively, the residual should be zero when transforming the scanned points ${\{}^{L}p_{j}\}$ in the LiDAR coordinate system L to the global coordinate system G through a pose change. From this, the geometric relationship between the LiDAR measurement points and the map planes can be derived as follows:

(7) 0=rl(xk,Lpj)=ujT(TIkGTLIpjL−qj)

#### 3.3.4. Visual Measurement Model

This study uses a direct method for processing visual information by minimizing the photometric error between the current image and the reference image patch to achieve image alignment. At $t_{k}$, the system receives a new camera scan frame and performs matching and searching on the global visual map to find map points ${\{}^{G}p_{i}\}$ that fall within the current frame. For these map points, the image patch in the global visual map with the closest viewing angle to the current image should be selected as the reference image patch $Q_{i}$.

During this process, for accurate image alignment, the photometric error between the reference image patch and the corresponding image patch in the current image should be minimized. This method achieves optimal matching between image patches by comparing and adjusting photometric differences pixel by pixel. The process of direct method image alignment can thus be derived as follows:

(8) $$0=r_{c}(x_{k}{,}^{G}p_{i})=I_{k}(\pi {(}^{I}T_{C}^{-1G}T_{I_{k}}^{-1G}p_{i}))-A_{i}Q_{i}$$

where π(∙) represents the pinhole camera model.

The direct method involves capturing the current frame with the camera and identifying potential matching points in the global map. Next, the reference image patch with the viewing angle closest to the current image is selected. Finally, the position and orientation of the image patches are adjusted by minimizing the photometric error until the error approaches zero, achieving accurate image alignment. This approach not only simplifies the computational process but also enhances the accuracy and efficiency of the alignment.

### 3.4. Error Iterative Kalman Filter Model

In the state estimation model, the prior distribution of the system state $x_{k}$ should satisfy the form of Equation (9):

(9) $$x_{k}⊞{\hat{x}}_{k}∼N(0,{\hat{P}}_{k})$$

Furthermore, the maximum a posteriori estimate of $x_{k}$ is obtained as

(10) $$\underset{x_{k}\in M}{\min}\left(∥x_{k}⊟{\hat{x}}_{k}{∥}_{{\hat{P}}_{k}}^{2}+\sum_{j=1}^{m_{l}}∥r_{l}(x_{k}{,}^{L}p_{j}){∥}_{\Sigma_{l}}^{2}+\sum_{i=1}^{m_{c}}∥r_{c}(x_{k}{,}^{G}p_{i}){∥}_{\Sigma_{c}}^{2}\right)$$

where $x_{\Sigma }^{2}=x^{T}\Sigma^{-1}x$ represents the weighted norm, measuring the magnitude of the state x under the covariance matrix.

## 4. Dynamic Removal

### 4.1. Principle of Dynamic Object Removal

Dynamic removal is divided into four stages: dynamic object detection, potential dynamic point detection, actual dynamic point detection, and dynamic point removal. Dynamic object detection mainly identifies dynamic objects in image frames using the lightweight neural network YOLOv5. In this study, for the complex urban environment, dynamic objects are set as vehicles and pedestrians. If there are dynamic pedestrians or vehicles in the image frame, dynamic recognition exports visual recognition areas ApV and AcV, respectively. These visual recognition areas are projected onto the corresponding LiDAR frame to obtain recognition areas ApL and AcL, respectively, on the LiDAR, and all points within these LiDAR recognition areas are considered potential dynamic points, which are stored in the form of an ikd-Tree [20].

For the potential dynamic points within a single LiDAR recognition area, this paper employs the Euclidean space clustering algorithm to identify the actual dynamic points. Euclidean space clustering is a hierarchical algorithm based on Euclidean distance, which groups points that are spatially close to each other. The clustering result is represented as a dendrogram, where each node corresponds to a cluster. The steps are as follows: First, all sample points are traversed, and a merge distance threshold ε is set. The Euclidean distance between two points x and y in an n-dimensional space is calculated, defined as d(x,y). During the traversal, if d(x,y)<ε, the points x and y are merged into the same cluster. This process is repeated until no further merging is possible. The advantage of the Euclidean space clustering algorithm lies in its simplicity and high efficiency when processing small-scale datasets. However, it is sensitive to noise and less efficient when handling large-scale data. In this study, the algorithm is applied to LiDAR recognition areas because the number of points requiring classification within each area is relatively small, making it well-suited for this efficient processing approach. Additionally, it meets the real-time requirements of dynamic odometry.

**Figure 3:**
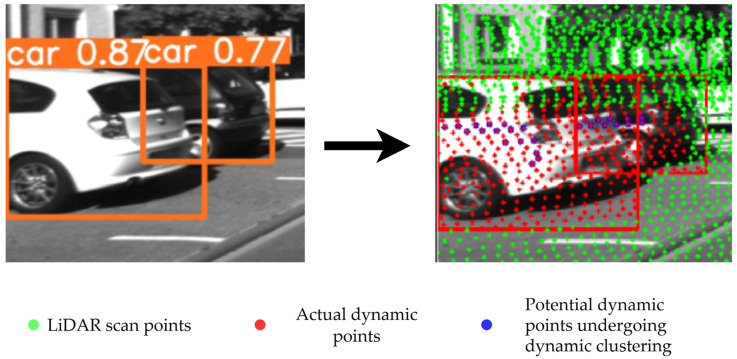
Dynamic removal diagram.

The steps of the Euclidean space clustering algorithm used for dynamic point removal are as follows: For potential dynamic points within a single LiDAR recognition area, Euclidean space clustering is used to identify the actual dynamic points. First, all potential dynamic points $D_{i}$ within a single LiDAR recognition area and their spatial coordinates $\left(x_{i},y_{i},z_{i}\right)$ are set. All potential dynamic points are traversed to identify the one closest to the center of the LiDAR recognition area, $D_{j}$. The merge distance threshold ε is set, and all dynamic points are traversed again. When $\sqrt{{\left(x_{i}-x_{j}\right)}^{2}+{\left(y_{i}-y_{j}\right)}^{2}+{\left(z_{i}-z_{j}\right)}^{2}}<\varepsilon$, $D_{i}$ is marked as the actual dynamic point and removed from the LiDAR stored data. After the traversal, the dynamic removal for that frame is completed, as shown in Figure 3. The orange rectangle represents the visual recognition area AcV, which identifies the specific type of dynamic object and provides confidence scores. The red rectangle denotes the LiDAR recognition area AcL. Green dots indicate LiDAR scan points within the current image frame, red dots represent identified dynamic points, and blue dots are potential dynamic points currently being converted through Euclidean space clustering. As shown in Figure 3, the dynamic removal method eliminates the main dynamic components of moving vehicles, leaving background points that can be used for mapping.

### 4.2. Dynamic Object Removal Strategy in Real-World Environments

In real urban environments, in addition to vehicles moving on the road, there are often stationary vehicles parked alongside the road. These stationary vehicles, like the road itself and the surrounding buildings, can provide accurate information for odometry. However, the information they provide is limited. Compared to flat road surfaces and building facades, static vehicles have a relatively minor impact on localization accuracy. If we attempt to differentiate between truly dynamic and static vehicles in real-world environments and only remove dynamic vehicles, the computational cost can increase significantly, posing a substantial burden on algorithms that already utilize lightweight neural networks.

Given these considerations, we removed all detected vehicles, both static and dynamic. Although this method sacrifices some information from static vehicles, it primarily achieves the goal of eliminating dynamic vehicles, which are the most impactful on real-world road mapping.

**Figure 4:**
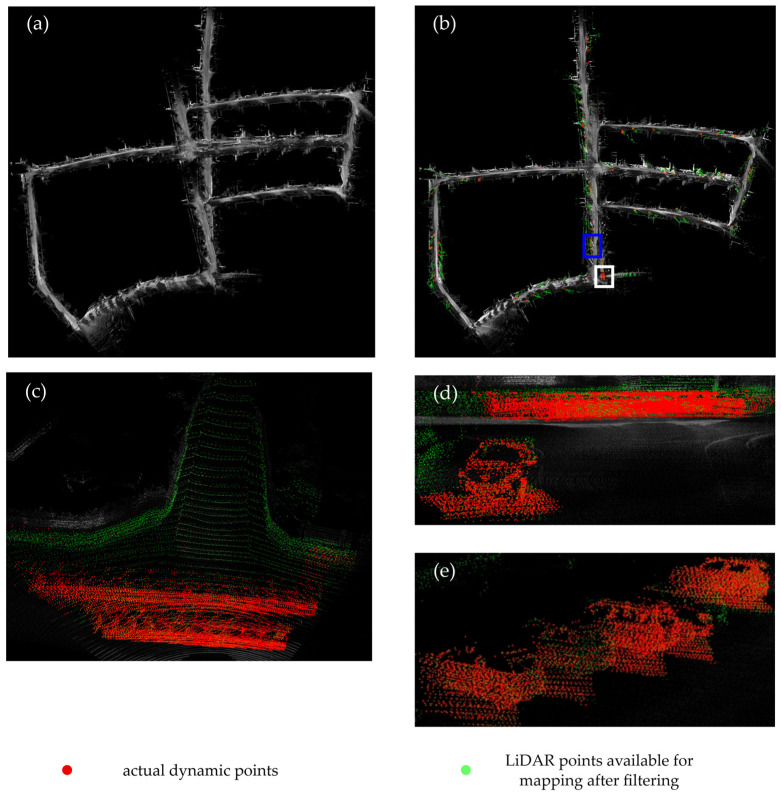
(**a**) Mapping results of FAST-LIVO, (**b**) mapping results of the improved algorithm, The blue square represents the removal area for static vehicles, and the white square represents the removal area for dynamic vehicles, (**c**) details of trailing effects, (**d**) Details of trailing effects from the driver’s perspective, (**e**) Enlarged view of the static vehicle removal area.

The results of subsequent simulation experiments confirm the feasibility of our strategy. As shown in Figure 4, when comparing the mapping performance of the FAST-LIVO algorithm with the improved dynamic odometry on sequence 05 of the KITTI dataset [21,22], we observe a noticeable improvement in the mapping quality of the middle of the road. The FAST-LIVO algorithm is significantly affected by dynamic vehicles when mapping around corners, resulting in substantial deviations when re-entering the middle of the road for the second time. In contrast, the improved dynamic odometry, after removing dynamic objects, shows a clear improvement when re-entering the middle of the road for the second time.

**Figure 5:**
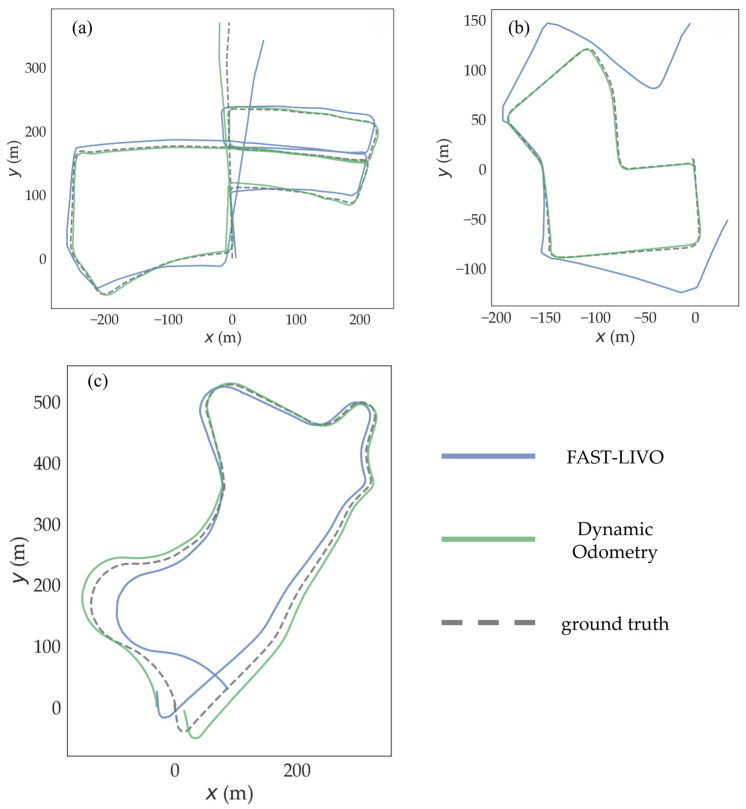
Comparison of global trajectory: (**a**) sequence 05, (**b**) sequence 07, and (**c**) sequence 09.

Figure 5a provides a more intuitive observation of the improvement in mapping accuracy from a global trajectory perspective. Before encountering dynamic vehicles around corners, the difference in the mapping accuracies between FAST-LIVO and the dynamic odometry is minimal. However, after encountering dynamic vehicles at corners, the dynamic odometry shows a significant improvement in mapping accuracy compared to FAST-LIVO.

## 5. Simulation Experiments

To validate the performance of the proposed dynamic odometry algorithm in both indoor and real-world dynamic environments, we conducted tests using the KITTI and NTU VIRAL datasets. The NTU VIRAL dataset, collected by a small drone, represents indoor environments. The KITTI dataset, collected by a car equipped with multiple sensors, represents real urban road dynamic environments. The simulations in this study were conducted on an Ubuntu 20.04 system with an Intel i7-1087H processor manufactured by Intel (USA) and an RTX 2060 GPU manufactured by NVIDIA (Santa Clara, CA, USA).

The data processing method used in this study was a post-processing approach based on offline simulation tests using publicly available datasets. The experimental data were collected by the dataset publishers on their self-built experimental platform. The dataset is stored in the form of “rosbag” and contains data from various sensors.

### 5.1. Real-World Urban Road Dynamic Environment Simulation Validation

The KITTI dataset [21,22] was collected using a platform that includes two grayscale cameras, two color cameras, one 3D LiDAR, and one GPS navigation system, which provides accurate ground-truth trajectories. The specific models and parameters of the sensors used were as follows: The grayscale and color cameras were PointGrey Flea2 manufactured by PointGrey (Richmond, BC, Canada) with a resolution of 1392 × 512 pixels, providing grayscale and color visual information at an actual publishing frequency of 10 Hz. The 3D LiDAR was the Velodyne HDL-64E manufactured by Velodyne (San Jose, CA, USA), with an effective range of up to 100 m, providing LiDAR point cloud data at an actual publishing frequency of 10 Hz. The dataset contains real-world image data from urban, rural, and highway scenes and includes dynamic vehicles and pedestrians, making it suitable for evaluating the algorithm proposed in this paper.

The dynamic odometry algorithm proposed in this paper was validated on sequences 05, 07, and 09 of the KITTI dataset. These sequences feature complex paths and a significantly dynamic environment. Figure 4 shows the mapping results of the proposed algorithm compared to the FAST-LIVO algorithm on sequence 05. The results reveal a noticeable improvement in mapping accuracy with the improved algorithm. 

In the figure, green dots represent LiDAR points used for mapping after removing dynamic points, while orange-red dots indicate the removed dynamic points. Figure 4e zooms in on the vehicle removal section within the blue box of Figure 4b, providing a detailed view of the process of vehicle detection and removal. In the zoomed-in view of Figure 4c, vehicles driving in the T-intersection area create trailing effects during mapping, and Figure 4d presents these trailing effects more intuitively from the driver’s perspective. Dynamic objects scanned by LiDAR are also perceived as continuous obstacles, resulting in the appearance of obstacles in the middle of the road, which severely affects the mapping accuracy of traditional algorithms. The improved algorithm removes these trailing effects and retains static LiDAR points for mapping, thereby enhancing mapping accuracy.

**Figure 6:**
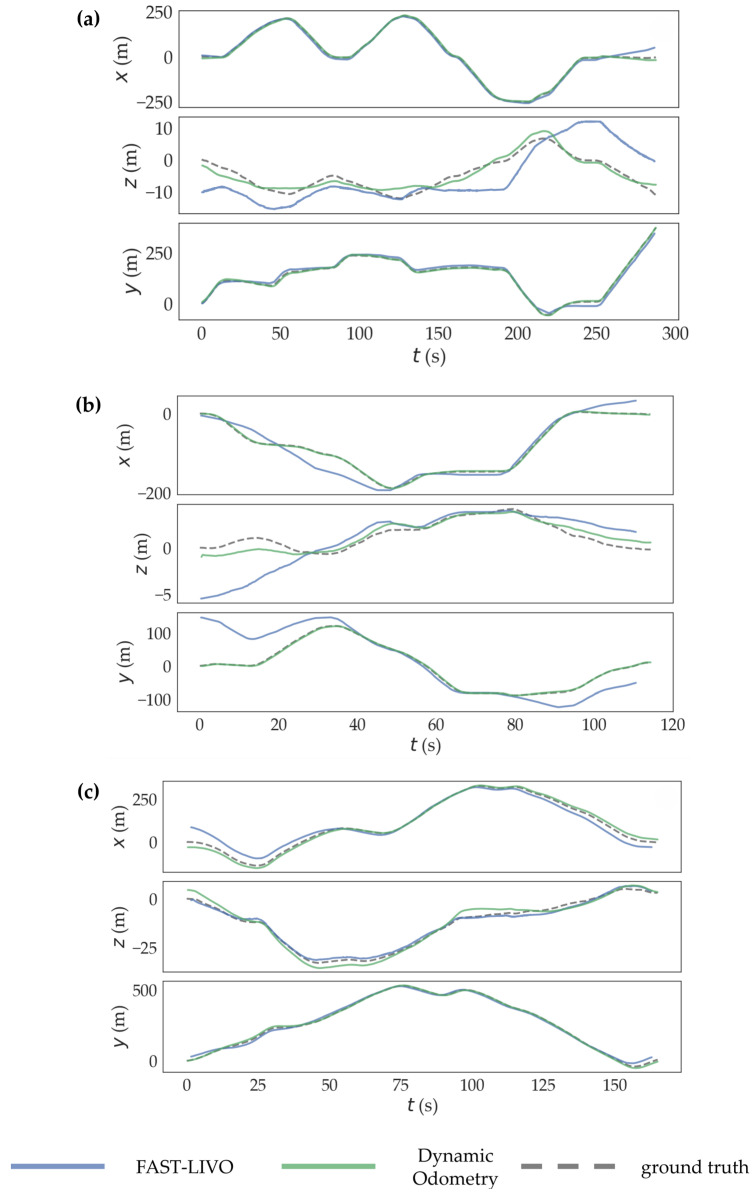
Comparison of axial trajectory: (**a**) sequence 05, (**b**) sequence 07, and (**c**) sequence 09.

A comparison of mapping accuracy between the improved algorithm and the FAST-LIVO algorithm on sequences 05, 07, and 09 is shown in Table 1. The evaluation metrics include the maximum distance error, minimum distance error, and RMSE. The global and axial trajectory comparisons are shown in Figure 5 and Figure 6. The comparison data indicate that the improved algorithm shows significantly better accuracy metrics for mapping. The original algorithm failed to map sequence 07, whereas the improved algorithm successfully mapped it with high accuracy. We define $\partial_{f}$ as the mapping RMSE of FAST-LIVO in the dataset sequences, $\partial_{d}$ as the mapping RMSE of our improved dynamic odometry in the dataset sequences, and the average RMSE reduction as $\frac{\left(\partial_{f}-\partial_{d}\right)}{\partial_{f}}$. The proposed method reduced the RMSE of mapping by 72.73% and 57.50% in sequences 05 and 09, respectively. For sequence 07, where the original algorithm failed to map, the improved algorithm achieved high mapping accuracy.

### 5.2. Indoor Small-Scale Environment Simulation Validation

The NTU VIRAL dataset [23] is a public dataset for indoor small-scale environments used for autonomous drone navigation. The data were collected by a small drone equipped with two 3D LiDARs, two hardware-synchronized global shutter cameras, multiple IMUs, and several ultra-wideband (UWB) ranging units, with the UWB providing precise pose information. The specific models and parameters of the sensors used are as follows: The IMU was the VectorNav VN1003 manufactured by VectorNav (Dallas, TX, USA) rugged IMU, which provides measurements of angular velocity and acceleration with an effective actual publishing frequency of up to 385 Hz. The 3D LiDAR was a 16-channel OS1 gen1 laser scanner, which provides LiDAR point cloud data with an actual publishing frequency of 10 Hz. The stereo camera was the uEye 1221 LE manufactured by IDS (Obersulm, Germany) monochrome global shutter camera, with a resolution of 752 × 480 pixels, providing visual information at an actual publishing frequency of 10 Hz. The UWB system was the Humatics P440 manufactured by Humatics (Waltham, MA, USA), with two anchor points set by the authors to measure the true trajectory of the drone and an actual publishing frequency of 10 Hz.

**Figure 7:**
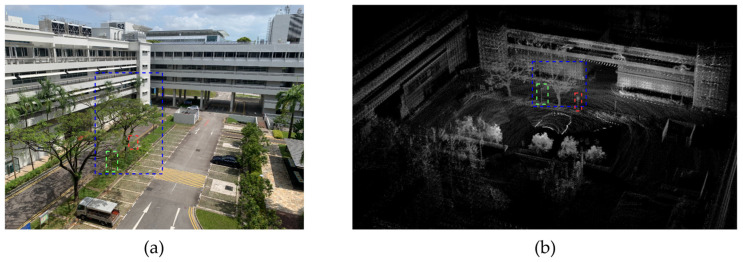
(**a**) Photo of the surrounding environment in the eee_03 sequence; (**b**) mapping results of the improved algorithm.

The dynamic odometry algorithm proposed in this study was validated on all sequences of the NTU VIRAL dataset, with results from the eee_03 sequence used as a specific example for detailed analysis. The blue box corresponds to the trees in the scene, while the green and red boxes correspond to the pillars in the scene. Figure 7 illustrates the mapping results of the proposed algorithm on the eee_03 sequence. The figure shows that the improved algorithm effectively captures details such as trees, building supports, and roads, providing a fairly complete reconstruction of the overall environment.

**Figure 8:**
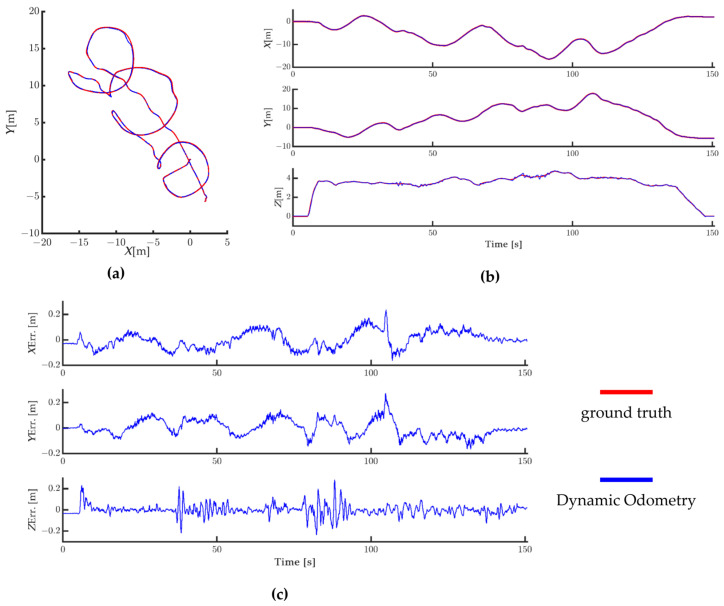
(**a**) Global trajectory comparison, (**b**) axial trajectory comparison, and (**c**) axial error variation.

Figure 8 shows the global trajectory, axial trajectory, and axial error variation of the improved algorithm on the eee_03 sequence. As depicted in Figure 8, the predicted global trajectory aligns closely with the ground truth with minimal error. In the axial trajectories, the *X*- and *Y*-axes closely match the ground truth with minimal error, while the *Z*-axis trajectory shows some slight jitter at 40 and 85 s. In the axial error variation plot, errors for all axes fluctuate within 0.2 m. The experimental results in Figure 8 indicate that the improved algorithm performs well in indoor small-scale mapping, maintaining high positioning accuracy even after several minutes of flight and after covering hundreds of meters.

Table 2 compares the mapping accuracy of the improved algorithm and FAST-LIVO across all sequences of the NTU VIRAL dataset, with the evaluation metric being the root mean square error (RMSE). As shown in the data from Table 2, although the NTU VIRAL dataset does not contain dynamic objects, the improved algorithm consistently outperforms FAST-LIVO in mapping accuracy across all sequences. This improvement indicates that packaging sensor data into computational units enhances positioning accuracy. By sorting the input data by time, the algorithm ensures that the IMU, image, and LiDAR data are processed more consistently, improving data synchronization and reducing errors caused by time delays. The mapping accuracy is successfully improved from the decimeter level to the centimeter level. 

The method for calculating the average RMSE reduction in this section is the same as in the previous section, with the average RMSE reduction being 60.29% in the eee01–03 sequences, 60.71% in the nya01–03 sequences, and 69.86% in the sbs01–03 sequences.

## 6. Conclusions

In this study, based on the FAST-LIVO algorithm, we devised a multi-sensor fusion dynamic odometry method using the YOLOv5 neural network. Our approach enhances the alignment in LIO by packaging sensor data into computational units, better leveraging the advantages of batch processing and parallel computing, thereby improving the real-time performance of the odometry. It ensures more consistent processing of IMU, image, and LiDAR data, enhancing data synchronization and reducing errors caused by time delays. In terms of dynamic removal, a lightweight neural network is utilized to remove dynamic points at the LiDAR level, classifying the dynamic points within the detection box as potential dynamic points and actual dynamic points. The actual dynamic points are removed, while the potential dynamic points are retained for mapping.

The proposed dynamic odometry method achieved better mapping results on both the KITTI and NTU VIRAL datasets, with improved mapping accuracy compared to the FAST-LIVO algorithm.

## Figures and Tables

**Table 1:** Comparison of mapping accuracy metrics.

Sequence	Algorithm	Max (m)	Min (m)	RMSE (m)
05	FAST-LIVO	58.67	5.61	19.17
	Dynamic odometry	15.50	0.32	5.23
07	FAST-LIVO	145.96	1.04	46.21
	Dynamic odometry	3.59	0.32	2.04
09	FAST-LIVO	89.59	1.13	31.78
	Dynamic odometry	31.06	1.08	13.50

**Table 2:** Comparison of RMSE on the NTU VIRAL dataset (m).

	eee_01	eee_02	eee_03	nya_01	nya_02	nya_03	sbs_01	sbs_02	sbs_03
FAST-LIVO	0.28	0.17	0.23	0.19	0.18	0.19	0.29	0.22	0.22
Dynamic odometry	0.10	0.07	0.10	0.06	0.08	0.08	0.08	0.07	0.07

## Data Availability

Data are contained within the article.
